# Microstructure and Mechanical Properties of TiC_0.7_N_0.3_-HfC-WC-Ni-Mo Cermet Tool Materials

**DOI:** 10.3390/ma11060968

**Published:** 2018-06-08

**Authors:** Jiaojiao Gao, Jinpeng Song, Ming Lv

**Affiliations:** 1 School of Mechanical Engineering, Taiyuan University of Technology, Taiyuan 030024, China; gaojiaojiao1207@163.com; 2 Shanxi Key Laboratory of Precision Machining, The Shanxi Science and Technology Department, Taiyuan University of Technology, Taiyuan 030024, China

**Keywords:** TiCN-HfC-WC cermet, hot-pressed sintering, microstructure, mechanical properties

## Abstract

TiC_0.7_N_0.3_-HfC-WC-Ni-Mo cermet tool materials were fabricated by hot pressing technology at 1450 °C. The effects of WC (tungsten carbide) content on the microstructure and mechanical properties of TiC_0.7_N_0.3_-HfC-WC-Ni-Mo cermet tool materials were investigated. The results showed that the TiC_0.7_N_0.3_-HfC-WC-Ni-Mo cermets were mainly composed of TiC_0.7_N_0.3_, Ni, and (Ti, Hf, W, Mo)(C, N); there were three phases: a dark phase, a gray phase, and a light gray phase. The dark phase was the undissolved TiC_0.7_N_0.3_, the gray phase was the solid solution (Ti, Hf, W, Mo)(C, N) poor in Hf, W, and Mo, and the light gray phase was the solid solution (Ti, Hf, W, Mo)(C, N) rich in Hf, W, and Mo. The increase of WC content could promote the process of HfC to form a solid solution and the HfC formed a solid solution more easily with WC than with TiCN. The increase of the solid solution made the microstructure more uniform and the mechanical properties better. In addition, the Vickers hardness, flexural strength, and fracture toughness of the TiC_0.7_N_0.3_-HfC-WC-Ni-Mo cermet increased with the increase of WC content. When the content of WC was 32 wt %, the cermet obtained the optimal comprehensive mechanical properties in this investigation. The toughening mechanism of TiC_0.7_N_0.3_-HfC-WC-Ni-Mo cermet tool materials included solid solution toughening, particle dispersion toughening, crack bridging, and crack deflection.

## 1. Introduction

TiCN-based cermet tool material has developed rapidly in recent years and exhibits excellent characteristics in high-speed cutting [1]. It has excellent properties such as good wear resistance, excellent high temperature hardness, a relatively low friction coefficient, and superior chemical stability [2,3]. Compared with the traditional WC-Co tool, the TiCN cermet tool has better thermal stability, high temperature oxidation resistance, high temperature hardness, and lower fracture toughness [4,5,6]. Recently, researchers have conducted a lot of research on how to improve the fracture toughness of TiCN cermet. Yin et al. [2] investigated the effect of the microwave sintering process and WC/Mo_2_C ratio on the mechanical properties of TiCN-based cermet cutting tool material, and reported that the fracture toughness was 10.08 MPa·m^1/2^ and the hardness was 16.83 GPa. Verma et al. [5] investigated the effect of TaC addition on the mechanical properties of Ti(CN)-5 wt % WC-20 wt % Ni/Co cermets and reported that the fracture toughness was 9.51 MPa·m^1/2^ and the hardness was 17.01 GPa. Liu et al. [1] investigated the influence of Mo_2_C and TaC additions on the mechanical properties of TiCN-based cermets, and pointed out that the fracture toughness was about 13.5 MPa·m^1/2^, the transverse rupture strength was about 1690 MPa, and the hardness was about 91.4 HRA. Park et al. [7] investigated carbide/binder interfaces in Ti(CN)-(Ti, W)C/(Ti, W)(CN)-based cermets and the result showed that the fracture toughness was 12.2 MPa·m^1/2^ and the hardness was 14 GPa. Sun et al. [8] investigated the effect of short carbon fiber concentration on the mechanical properties of TiCN-based cermets and stated that the fracture toughness was 11.66 MPa·m^1/2^, the bending strength was 643.3 MPa, and the hardness was 79.1 HRA. It can be noted that there has been a great breakthrough in improving the fracture toughness of TiCN-based cermet, but its hardness has not improved simultaneously. Its low hardness limits its extensive application in high-speed cutting. Therefore, it is necessary to improve the comprehensive mechanical properties of TiCN-based cermet.

In many investigations, WC plays an important role in enhancing the microstructure and mechanical properties of TiCN-based cermet. Dong et al. [9] reported that the mechanical properties of nano Ti(C, N)-based cermet were improved with the increase of WC addition. Qu et al. [10] reported that the lattice constant of the Ti(C, N) hard phase increased with the increase of WC content. Wang et al. [11] reported that WC can enhance density, facilitate sintering, and decrease the particle growth rate of TiCN-based cermet, and that Mo is almost necessary for Ti(CN)-based cermets. However, there are few reports on the effect of WC on the microstructure and mechanical properties of TiCN-HfC cermet. Besides, Mun et al. [12] reported that the addition of HfC could improve the cutting performance of TiCN-based cermet tools and Song et al. [13] pointed out that HfC addition could inhibit grain growth in TiN-based and TiB_2_-based ceramic tools. Based on our previous investigation [14], HfC plays a positive role in improving the microstructure and mechanical properties of TiCN-based cermet tools. In order to further improve the comprehensive mechanical properties of TiCN-HfC cermet, the effect of WC content on the microstructure and mechanical properties of TiCN-HfC-Ni-Mo cremet was investigated in this work.

## 2. Experimental Procedure

The composition of TiC_0.7_N_0.3_-HfC-WC-Ni-Mo cermets and the specifications of raw powders are showed in Table 1 and Table 2.

The powders were mixed and milled for 48 h in a polyethylene jar with WC (tungsten carbide) balls and alcohol as mediums. Then the mixed slurry was dried in vacuum and sieved by a 200-mesh sieve. Then, the mixed powder was sealed and compacted into graphite mold at room. The compacted powders were sintered in a vacuum ((1.2−2.4) × 10^−3^ Pa) hot-press sintering furnace (ZT-40-20, Shanghai Chenhua Technology Co., Ltd., Shanghai, China). The sintering parameters were as follows: the sintering temperature was 1450 °C, the holding time was 30 min, the heating rate was 50 °C/min, and the sintering pressure was 30 MPa. The sintered samples were cut into testing specimens by the electrical discharge wire cutting method and the surfaces of the testing bars were polished using diamond slurries. The dimensions of the specimen were 3 mm × 4 mm × 40 mm.

Flexural strength was measured at a span of 30 mm and across a head speed of 0.5 mm/min by the three-point bending test method on an electron universal tester (CREE-8003G, Dongguan City Kerry Instrument Technology Co., Ltd., Dongguan, China) according to Chinese National Standards GB/T 6569-2006/ISO 14704: 2000 [15]. Fracture toughness (*K_IC_*) was measured via the direct indentation method [16,17]. Vickers hardness was measured on the polished surfaces using a diamond pyramid indenter (HVS-30, Shanghai Precision Instruments Co., Ltd., Shanghai, China) under a load of 196 N for 15 s by HV-120 based on Chinese National Standards GB/T 16534-2009 [18]. The relative density of each specimen was measured by the Archimedes method with distilled water as the medium. The theoretical density was calculated according to the rule of mixtures based on the following densities: 5.08, 12.7, 15.6, 8.90, and 10.20 g/cm^3^ for TiC_0.7_N_0.3_, HfC, WC, Ni, and Mo, respectively. At least 15 specimens were tested for each experimental condition. X-ray diffraction (XRD, EMPYREAN, PANalytical B.V., Almelo, The Netherlands) and energy dispersive spectrometry (EDS, ACT-350, Oxford Instruments, Oxford, UK) were used to analyze the compositions of the composite. A scanning electron microscope and a back-scattered electron microscope (SEM, Supra-55, Carl Zeiss AG, Oberkochen, Germany) were used to observe the polished surface and fractured surface morphologies.

## 3. Results and Discussion

### 3.1. Effects of WC Content on the Microstructure of TiC_0.7_N_0.3_-HfC-WC-Ni-Mo Cermets

**Figure 1:**
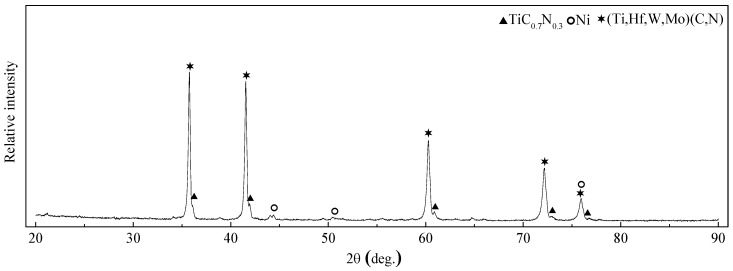
R-ray diffraction (XRD) patterns of TiC_0.7_N_0.3_-HfC-WC-Ni-Mo cermet tool materials.

Figure 1 exhibits the XRD patterns of TiC_0.7_N_0.3_-HfC-WC-Ni-Mo cermet tool materials. The main phases were TiC_0.7_N_0.3_, Ni and (Ti, Hf, W, Mo)(C, N). The solid solution (Ti, Hf, W, Mo)(C, N) had a similar lattice structure to that of TiC_0.7_N_0.3_ and solid solutions similar to that of (Ti, W/Ta/Mo…)(C, N) were also found in other researches [1,5,11]. In the liquid-phase sintering stage, TiC_0.7_N_0.3_, WC, HfC, and Mo dissolved, and then precipitated as a complex solid solution, (Ti, Hf, W, Mo)(C, N).

**Figure 2:**
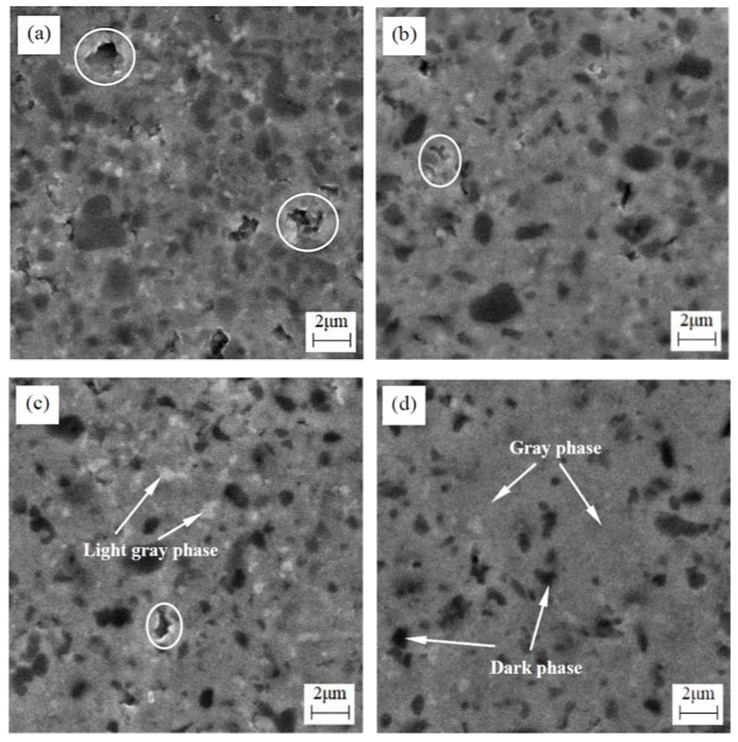
SEM micrographs of polished surfaces of TiC_0.7_N_0.3_-HfC-WC-Ni-Mo cermet tool materials with different WC contents: (**a**) TiC_0.7_N_0.3_-HfC-8 wt % WC (W8); (**b**) TiC_0.7_N_0.3_-HfC-16 wt % WC (W16); (**c**) TiC_0.7_N_0.3_-HfC-24 wt % WC (W24); (**d**) TiC_0.7_N_0.3_-HfC-32 wt % WC (W32).

**Figure 3:**
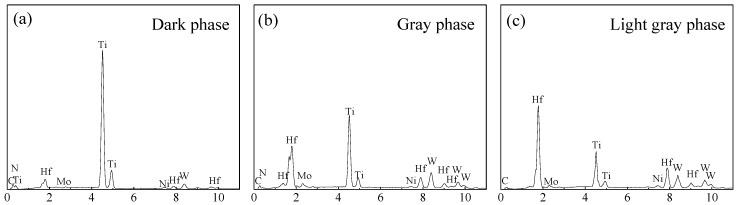
Energy dispersive spectrometry (EDS) results of the phases in TiC_0.7_N_0.3_-HfC-WC-Ni-Mo cermet tool materials: (**a**) EDS results of the dark phase; (**b**) EDS results of the gray phase; (**c**) EDS results of the light gray phase.

Figure 2 shows the SEM micrographs of polished surfaces of TiC_0.7_N_0.3_-HfC-WC-Ni-Mo cermet tool materials with different WC contents. There were three phases including a dark phase, a gray phase, and a light gray phase, as shown by the arrows in Figure 2. The EDS result of each phase is exhibited in Figure 3. Based on the results of the XRD and EDS, the dark phase was the remanent TiCN with abundant Ti and scanty W, Hf, and Mo, as shown in Figure 3a; the gray phase was the complex solid solution (Ti, Hf, W, Mo)(C, N) poor in W, Hf, Mo and rich in Ti, as shown in Figure 3b; and the light gray phase was the complex solid solution (Ti, Hf, W, Mo)(C, N) rich in W, Hf, Mo elements but poor in Ti, as shown in Figure 3c. Qu et al. [10] reported that WC and Mo dissolved and then reprecipitated as solid solutions (Ti, W)(C, N) and (Ti, Mo)(C, N) in the Ti(C, N)-WC-Mo-Ni cermet, respectively; Mun et al. [12] reported that the solid solution (Ti, Hf)C formed in the dissolution-reprecipitation process in the TiC-HfC-Ni cermet; Kumar et al. [19] reported that (Ti, W/Nb/Ta/Hf)CN formed and reprecipitated around the undissolved TiCN in the TiCN-based cermets; therefore, the solid solution (Ti, Hf, W, Mo)(C, N) resulted from the dissolution-reprecipitation process. Zhou et al. [20] reported that the solid solution (Mo, Ti)(C, N) can improve the interface bonding strength between Ti(C, N) and Ni. In Figure 2 with the WC content increased from 8 to 32 wt %, the change in the size of the dark phase was not obvious, which was in the range of 0.5–3 μm, but the number of the dark phase decreased gradually, which indicated that the higher content of WC promoted the dissolution-reprecipitation process. In addition, there were some flaws (marked by circles) in Figure 2 and the flaw mostly presented in the light gray phase, which showed that the light gray phase was a weak phase. With the WC content increasing, the number of flaws decreased gradually. In particular, when the WC content increased to 32 wt %, there were almost no flaws in Figure 2d, which indicated that the quality of the polished surface was improved. The shapes of the flaws showed that the formation of flaws was mainly due to the pull-off of the grains during the grinding and polishing process [13], which showed that the grain bonding strength was weak. So the improved quality of the polished surface indicated that the grains bonding strength increased with the WC content increasing. The main reason for this was that the wettability of Ni to WC was very good, far superior to the wettability of Ni to TiCN and HfC. Therefore, with the WC content increasing, the number of flaws decreased gradually. The improvement of the grain bonding strength can restrain crack propagation and then improve the fracture toughness of the cermet. Apart from the removed grains, some pores probably forming in the sintering process were parts of the flaws.

**Figure 4:**
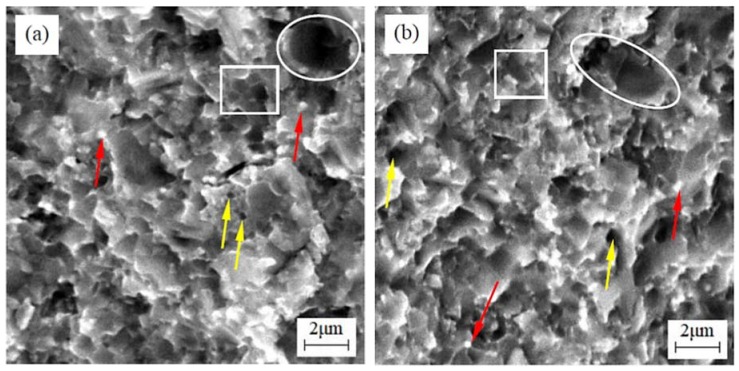

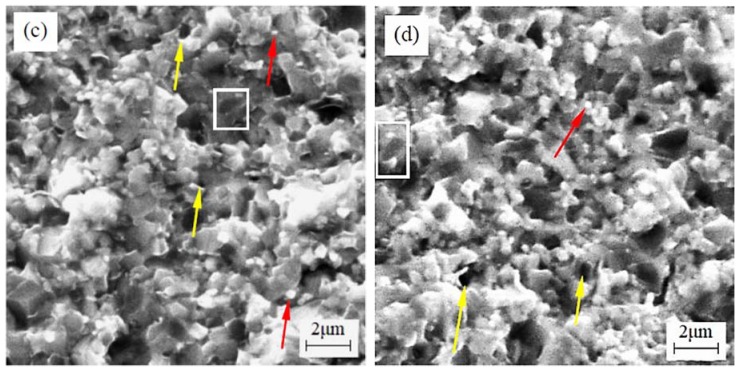
Fracture morphologies of TiC_0.7_N_0.3_-HfC-WC-Ni-Mo cermet tool materials with different WC contents: (**a**) TiC_0.7_N_0.3_-HfC-8 wt % WC (W8); (**b**) TiC_0.7_N_0.3_-HfC-16 wt % WC (W16); (**c**) TiC_0.7_N_0.3_-HfC-24 wt % WC (W24); (**d**) TiC_0.7_N_0.3_-HfC-32 wt % WC (W32).

Figure 4 shows the fracture morphologies of TiC_0.7_N_0.3_-HfC-WC-Ni-Mo cermet tool materials with different WC contents. The core-rim structure (marked by squares) was obvious in these cermets. During the sintering, HfC, WC, and Mo dissolved and then reprecipitated as the solid solution (Ti, Hf, W, Mo)(C, N) to form the rim around the core—undissolved TiCN. When the WC content was 8 and 16 wt %, there were some big size dark phases (marked by circles in Figure 4a,b) which made the microstructure uneven. In contrast, the microstructure was fine and uniform in Figure 4c,d when the WC content was 24 and 32 wt %, respectively. The main reason for this was that as the WC content increased, the rim tended to be complete, which isolated the direct contact of the TiCN and, in turn, inhibited the growth of TiCN. In addition, there were some white dots on the fracture surfaces (marked by the red arrows in Figure 4) and they decreased gradually as the WC content increased. Especially when the WC content was 32 wt % (in Figure 4d), the white dots were very few, which could also be validated in Figure 2d. According to our previous finding [14], the white dots were HfC particles. Therefore, these indicated that the increase of WC content could promote the process of HfC to form a solid solution and that HfC formed a solid solution more easily with WC than with TiCN. Besides, HfC particles locating at grain boundaries can play a very good pinning role for improving the mechanical properties of the cermet. In addition, there were some pores (marked by the yellow arrows in Figure 4) in the fracture surface. These pores were composed of the micro-void left by the pull-out grains in the fracture process and the pore formation in the sintering process. The pull-out effect of grains was advantageous to improve the mechanical properties of cermets, while the pore formation in the sintering process was harmful to the mechanical properties of cermets.

### 3.2. Effects of WC Content on the Relative Density and Mechanical Properties of TiC_0.7_N_0.3_-HfC-WC-Ni-Mo Cermets

**Figure 5:**
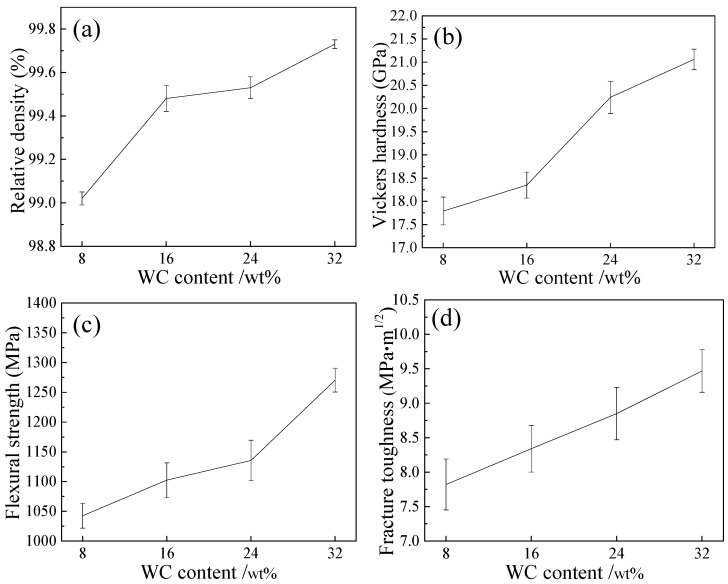
Relative density and mechanical properties of TiC_0.7_N_0.3_-HfC-WC-Ni-Mo cermet tool materials with different WC contents: (**a**) relative density; (**b**) Vickers hardness; (**c**) flexural strength; (**d**) fracture toughness.

Figure 5 presents the relative density and mechanical properties of TiC_0.7_N_0.3_-HfC-WC-Ni-Mo cermet tool materials with different WC contents. With the WC content increasing from 8 to 32 wt %, the relative density increased from (99.02 ± 0.03)% to (99.73 ± 0.02)%, the Vickers hardness increased from 17.79 ± 0.30 to 21.06 ± 0.22 GPa, the flexural strength increased from 1042.61 ± 21 to 1270.56 ± 20 MPa, and the fracture toughness increased from 7.82 ± 0.37 to 9.47 ± 0.31 MPa·m^1/2^. The improvement of the relative density and Vickers hardness was mainly due to the wettability of Ni between WC being much better than that of Ni between TiCN. Better wettability can improve the sintering property of the material, so better densification and hardness were obtained. Therefore, the higher the content of WC was, the higher relative density and hardness of the material was. Besides, the reduction of flaws promoted the improvement of the relative density of materials. In addition, the fine microstructure was also benefit for the enhancement of hardness. W32 had the best hardness of 21.06 ± 0.22 GPa, which was higher than the value of 19.4 GPa reported in TiC_0.7_N_0.3_-20 wt % HfC-Ni-Co cermet [14] and higher than the value of 17.54 GPa reported in TiCN-WC-Mo_2_C-Ni-Mo cermet [2].

For the flexural strength, it increased with the increase of WC content, which can be explained by the Griffith-Orowan equation [21]:

$$\sigma =(2EP/\pi L{)}^{1/2}$$

where *E*, *P*, and *L* represent the Young’s modulus, plastic deformation work due to crack stretching, and the length of the crack, respectively. The Young’s modulus values of TiC, TiN, HfC, and WC are 510, 350, 470, and 731 GPa [22], respectively. *E*_(WC)_ is much greater than *E*_(TiC)_, *E*_(TiN)_, and *E*_(HfC)_. Therefore, the flexural strength increased with the increase of WC content. Meanwhile, the improved flexural strength also benefited from the increase of grain bonding strength and the decrease of the weak light gray phase. When the material was subjected to external forces, the material with higher grain bonding strength would easily succumb to transgranular fracture. At this point, more fracture energy would be consumed. Besides, the increase of the solid solution was also advantage to the flexural strength. W32 had the best flexural strength of 1270.56 ± 20 MPa, which was higher than the value of 1018.04 MPa reported in TiC_0.7_N_0.3_-20 wt % HfC-Ni-Co cermet [14].

**Figure 6:**
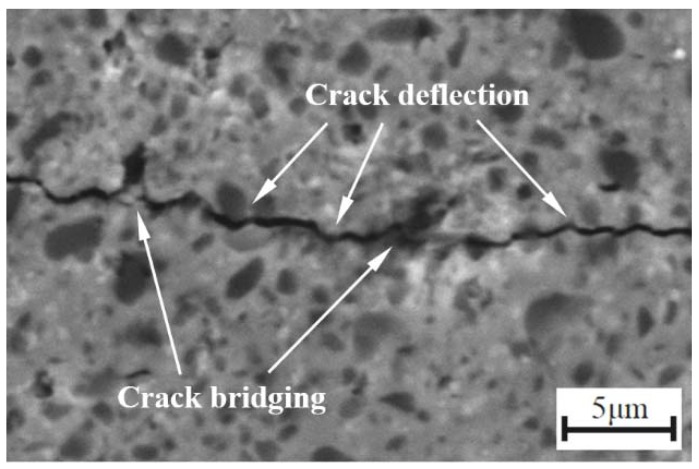
SEM micrograph of crack propagation of TiC_0.7_N_0.3_-HfC-WC-Ni-Mo cermet tool materials.

As for fracture toughness, it increased with the increase of WC content. The solubility of WC in Ni is higher than that of TiC in Ni [22]. The higher solubility was favorable for the toughness of the material. Also, the increase of grain bonding strength could efficiently inhibit crack propagation, so the fracture toughness increased. In addition, the uneven distribution of grains (as shown in Figure 4a,b) lead to the lower fracture toughness of W8 and W16 cermets. The reduction of the weak light gray phase was also advantageous to the improvement of the fracture toughness. W32 had the best fracture toughness of 9.47 ± 0.31 MPa·m^1/2^, which was higher than the value of 8.6 MPa·m^1/2^ reported in TiC_0.7_N_0.3_-20 wt % HfC-Ni-Co cermet [14], indicating that the formation of a mass of solid solution can improve the fracture toughness. In order to further determine the toughening mechanisms that operated in TiC_0.7_N_0.3_-HfC-WC-Ni-Mo cermet tool material, an SEM micrograph of the crack propagation path is exhibited in Figure 6. There were mainly crack deflection and crack bridging in TiC_0.7_N_0.3_-HfC-WC-Ni-Mo cermet tool materials. In the process of crack propagation, crack deflection and crack bridging can consume more crack propagation energy, so the crack deflection and crack bridging were advantageous to the improvement of the fracture toughness of the cermet.

## 4. Conclusions

TiC_0.7_N_0.3_-HfC-WC-Ni-Mo cermet tool materials with different WC content were sintered at 1450 °C by hot pressing sintering technology. Effects of WC content on the microstructure and mechanical properties of TiC_0.7_N_0.3_-HfC-WC-Ni-Mo cermet tool materials were investigated. The conclusions were as follows:

(1) TiC_0.7_N_0.3_-HfC-WC-Ni-Mo cermets were mainly composed of TiC_0.7_N_0.3_, Ni, and (Ti, Hf, W, Mo)(C, N). There were three phases: a dark phase, a gray phase, and a light gray phase. The dark phase was the undissolved TiC_0.7_N_0.3_, the gray phase was the solid solution (Ti, Hf, W, Mo)(C, N) poor in Hf, W, and Mo, and the light gray phase was the solid solution (Ti, Hf, W, Mo)(C, N) rich in Hf, W, and Mo. The solid solution (Ti, Hf, W, Mo)(C, N) resulted from the dissolution-reprecipitation process.
(2) With the WC content increasing, the grain bonding strength increased, which can restrain the crack propagation and then improve the fracture toughness of the cermet. In addition, the increase of WC content could promote the process of HfC to form a solid solution. Also, the HfC formed a solid solution more easily with WC than with TiCN.
(3) The relative density, hardness, flexural strength, and fracture toughness of the TiC_0.7_N_0.3_-HfC-WC-Ni-Mo cermet tool materials increased with the increase of the WC content in this investigation. When the content of WC was 32 wt %, the cermet obtained the optimal relative density and comprehensive mechanical properties: their values were (99.73 ± 0.02)%, 21.06 ± 0.22 GPa, 1270.56 ± 20 MPa, and 9.47 ± 0.31 MPa·m^1/2^, respectively.

## Figures and Tables

**Table 1:** Composition ratios of TiC_0.7_N_0.3_-HfC-WC-Ni-Mo cermets (wt %).

Specimen	TiC_0.7_N_0.3_	HfC	WC	Ni	Mo
W8	64	20	8	4	4
W16	56	20	16	4	4
W24	48	20	24	4	4
W32	40	20	32	4	4

**Table 2:** Specifications of raw powders.

Powders	Size	Purity	Manufacturer
TiC_0.7_N_0.3_	1 μm	>99%	Shanghai ST-Nano Science and Technology Co., Ltd., Shanghai, China
HfC	0.8 μm	>99%	Shanghai Chaowei Nanomaterials Co., Ltd., Shanghai, China
WC	0.1 μm	>99%	Shanghai Yunfu Nanotechnology Co., Ltd., Shanghai, China
Ni	1 μm	>99%	Shanghai Yunfu Nanotechnology Co., Ltd., Shanghai, China
Mo	1 μm	>99%	Shanghai Yunfu Nanotechnology Co., Ltd., Shanghai, China
